# A multi-country assessment of factors related to smallholder food security in varying rainfall conditions

**DOI:** 10.1038/s41598-017-16282-9

**Published:** 2017-11-24

**Authors:** Meredith T. Niles, Molly E. Brown

**Affiliations:** 1 Department of Nutrition and Food Sciences, Food Systems Program, 109 Carrigan Avenue, 350 Carrigan Wing, University of Vermont, Burlington, VT 05405 USA; 2 Department of Geographical Sciences, University of Maryland, 2181 LeFrak Hall, MD 20771, 703-855-6190 College Park, USA

## Abstract

Given that smallholder farmers are frequently food insecure and rely significantly on rain-fed agriculture, it is critical to examine climate variability and food insecurity. We utilize data from smallholder farmer surveys from 12 countries with 30 years of rainfall data to examine how rainfall variability and household resources are correlated with food security. We find that on average, households that experienced a drier than average year are 3.81 months food insecure, while households within a normal range of rainfall were 3.67 months food insecure, and wetter than average households were 2.86 months food insecure. Reduced odds of food insecurity is associated with agricultural inputs, ownership of livestock, water use efficiency, financial services, and participation in a group. However, in drier than average households, financial services as compared to agricultural inputs and agroecological practices have a greater prevalence of reduced instances of food insecurity, while agricultural inputs are more common for reduced food insecurity in wetter than average households. Only the use of fertilizer consistently results in reduced odds of food insecurity across all households regardless of rainfall, demonstrating that one-size fits all approaches to food security interventions are likely ineffective, and place-specific interventions considering climatic factors are critically important.

## Introduction

Climate variability, either through changes in temperature or precipitation, will have substantial impacts on biological systems and the smallholders, communities and countries that depend on them^1^. Food production is particularly vulnerable to climate because rainfall and temperature are the main drivers of crop growth and can also affect livestock production. Simultaneously, diseases and pest infestations, as well as water availability, affect the total amount of food produced in a region^2^. Under future projections, it is estimated that climate changes will create novel climates around the globe^3^ and significantly impact agricultural production while the demand for food increases^4^. As a result, a growing body of work aims to assess how farmers may adapt to such changes, and what factors may enable coping capacity and resilience to safeguard food security and household livelihoods.

There are an estimated 460–500 million smallholder farmers in the world who grow most of the food consumed in low-income countries^5^. These smallholder farmers have limited resource endowments, including small fields, only household members for labor, and minimal education, training, and finance to enable the adoption of new technologies^6,7^. As a result, climate change is a particularly important risk factor for smallholder farmers in the tropics, who are slated to be the most heavily affected and may lack many strategies for adaptation^8^. Given that the majority of smallholder farmers still rely on rain-fed agriculture^9^, and that the intensity and distribution of rainfall events are already changing in ways that affect farming (e.g. increased intensity, earlier or later season)^10–12^, the likely increase in climate and inter-annual rainfall variability will have profound consequences for smallholder farmers^13,14^.

Smallholder farmers who lack the capacity to invest in agricultural best management practices and technologies to achieve productivity gains are not able to produce more than they consume throughout the year, and many are net buyers of the crops that they produce^15^. The inability of smallholder farmers to participate in growing regional and urban markets can keep welfare gains to a minimum^16^. These challenges are further confounded when smallholders experience significant inter-seasonal and inter-annual variability in rainfall with food production impacts, which may result in steep local food price increases^17,18^. Given that most food is still produced and consumed locally in low income countries, projected changes to climate and rainfall variability present profound potential impacts for overall food security in these regions^19^.

However, the ability to ensure consistent smallholder farmer household food security is also influenced by complex factors including agricultural management of farming systems, the social capital of the household as it relates to the community, and a host of household characteristics such as the whether it is female-headed and education level^20^. Evidence suggests that agricultural interventions built on productivity-enhancing agricultural technologies (quality fertilizers, better seed varieties, improved livestock, and micro-irrigation), yielded 80–140% increases in income, significantly higher than investing in other parts of the agriculture value chain^21^. Social capital is also important to increase food security through farmer to farmer knowledge, information access, and external support^22^. Access to financial capital is also important for increasing adaptive capacity to climate shocks and change among smallholders, and is positively associated with market access^23^. These aforementioned interventions provide clear strategies that could help safeguard household food security and rural incomes, though it is not well understood how these different strategies may work in variable climate conditions.

Despite existing research, there is yet to be an exploration that combines climatic variables such as empirical rainfall data with household, financial resources and livelihoods data across multiple countries to examine their relationship to food security. Previous research connecting climate variability to food insecurity has found connections with poor nutrition outcomes for children based on the Demographic and Health Survey (DHS) data^13^. Anomalies of vegetation greenness were shown to have positive association with child survival and nutrition in four West African countries with a wide distribution of crop productivity^14^. Environmental factors can be important for child survival: in Burkina Faso and Mali, increased productivity reduced the probability of mortality, and in Mali, increased productivity is also associated with decreased odds of being severely wasted. More specific research related to rainfall has found that rainfall variability and food security are closely related^19,20,24^, though the impact of rainfall variability can fluctuate significantly based on household coping capacity^25^.

**Figure 1:**
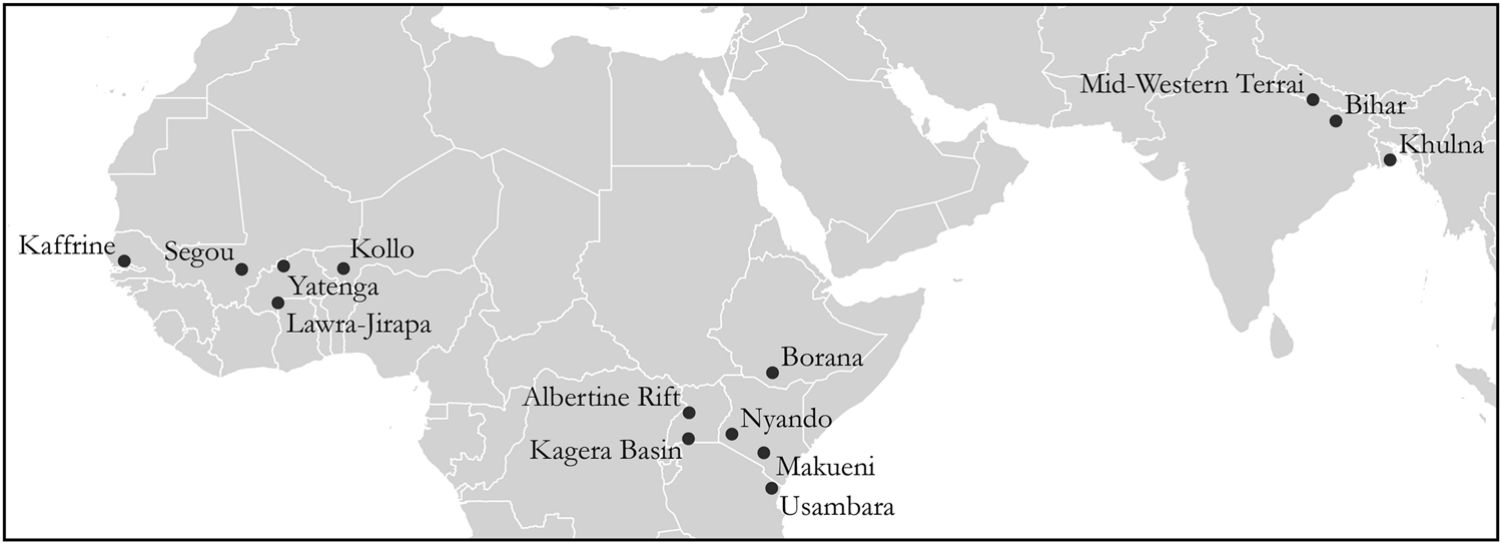
Locations of study sites. These 14 sites represent 12 countries in West and East Africa and South Asia across a gradient of rainfall variability. Figure generated using QGIS (2.16.3) (http://www.qgis.org/en/site/forusers/download.html#) and Adobe Illustrator CS5 (https://www.adobe.com/products/illustrator.html).

Here we combine 30 years of rainfall data with household survey data of smallholder farmers from the Climate Change, Agriculture, and Food Security (CCAFS) program across 14 sites and 12 countries to examine the relationship of climate, agricultural, financial, social, and household factors on food security outcomes in different rainfall conditions (Fig. 1). Unlike the DHS survey, the CCAFS survey includes data on demographic, financial and agricultural technologies, enabling more advanced analyses. Though the CCAFS dataset has been used in other analyses, these have focused on the relationship of food security to farm practice change in East Africa^26^, cropping decisions related to climate variability^27^, gender and climate resilience^28^, links between perceived climate shocks (i.e. drought) and food security (Niles and Salerno, submitted) and weather variability and food security in India only^29^.

Though these existing studies provide a foundation, they are limited in that they haven’t examined multiple livelihood strategies^30^, have taken a case study approach that limits broader application^26,29^, consider projected estimated rainfall changes rather than used actual rainfall data^4^, and have generally not used biophysical data in tandem with household surveys or examined food security specifically. The analysis presented in this paper enables an exploration for how different potential interventions across diverse places and rainfall gradients are correlated with household food security. This is important to assist a diversity of global health, agriculture and research organizations working globally to achieve scalea ble strategies for achieving food security in a changing climate.

## Results

### Food Insecurity and Rainfall Change

**Figure 2:**
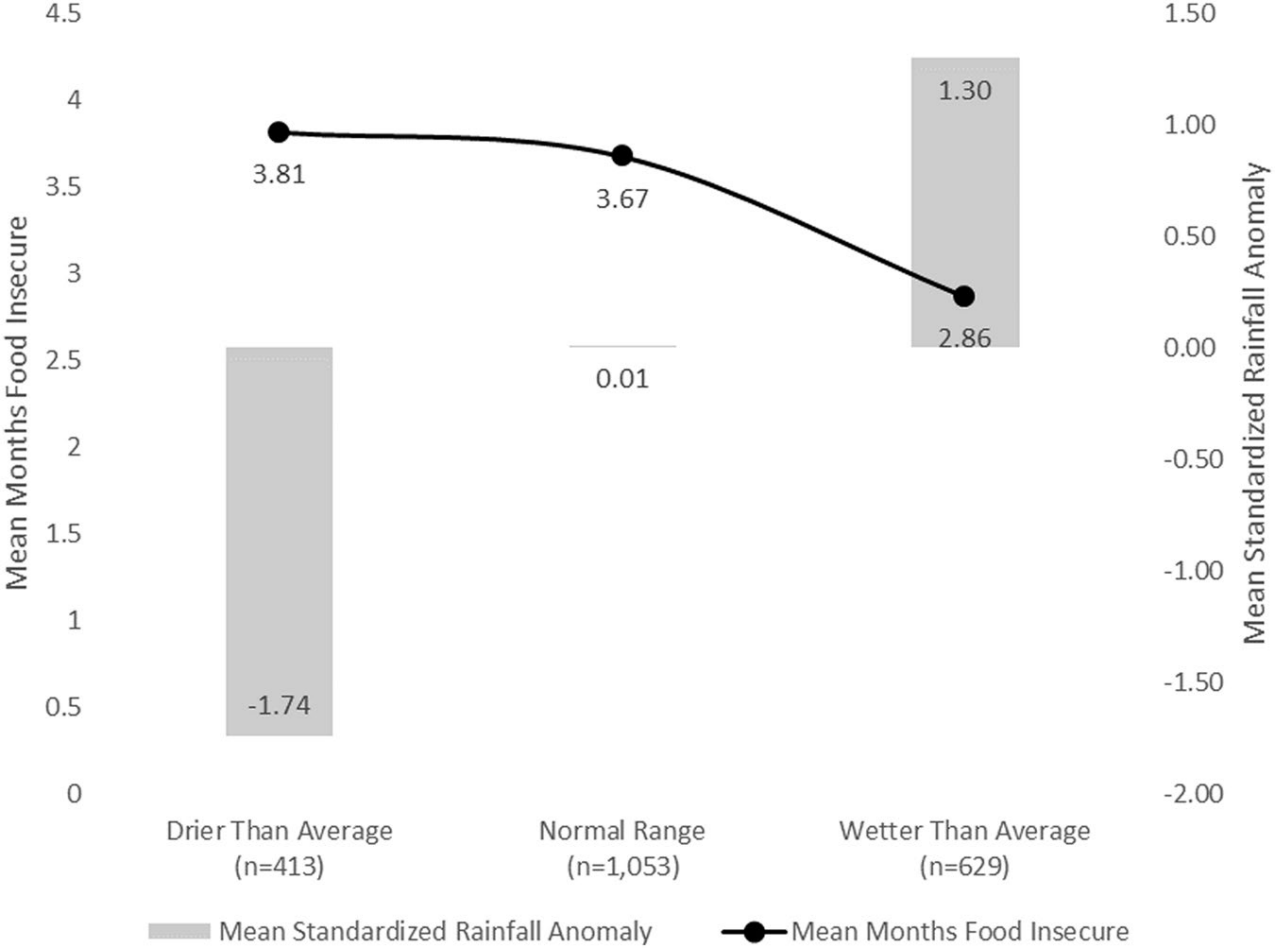
Mean standardized rainfall anomaly and mean months of food insecurity based on household type. Drier than average households experienced significantly less rainfall than the 30-year average and had significantly (*p* < 0.000) higher food insecurity compared with households that experienced wetter than average conditions.

We find that food insecurity is widespread across the global dataset with 80% of households experiencing at least one month of food insecurity. Average number of months of food insecurity (ranging from 0 to 12 months) in the previous year (12 months) prior to the survey varied significantly between drier than average households and wetter than average households (*p* < 0.000). Drier than average households had a mean 3.81 months food insecurity while wetter than average households had a mean of 2.86 months food insecurity. Households within the normal range were also significantly more likely to be food insecure (mean = 3.67) compared with wetter than average households (*p* < 0.000). Among household types, drier than average households had a mean rainfall anomaly of −1.74 standard deviations below the mean compared to normal range households mean of 0.01 and wetter than average households 1.30 standardized deviations above the mean (Fig. 2).

### Model and ANOVA Results

We ran 20 multi-level hierarchical random effects models to assess how different factors and rainfall anomaly on their own, and in interaction, predict food insecurity outcomes. Below we report the log odds of the factor main effect, rainfall anomaly, and the interaction effects as they relate to food insecurity (Table 1). In Supplementary Materials, we present figures showing the slope of a given factor across rainfall anomalies (Supplementary Figures 2–4). Further, since we wanted to explore how these factors may be correlated with different food insecurity outcomes, we also present ANOVA results, with the mean levels of food insecurity across household rainfall types by factors.

**Table 1:** Model Log Odds from 20 hierarchical random effects models. Main effects, rainfall effect, and interaction effects are reported here in log odds with p values.

Factor Type	Factors	Main Factor Effect	*p* =	Rainfall Effect (Absence of Factor)	*p* =	Interaction Effect (Factor with Rainfall)	*p* =
Agricultural Inputs	Certified Seed	**1.223**	***0.004***	**0.900**	***0.067***	1.002	*0.969*
	Fertilizer	**0.797**	***0.004***	0.935	*0.302*	0.995	*0.940*
	Manure	1.071	*0.365*	**0.855**	***0.008***	**1.136**	***0.031***
	Pesticides	**0.787**	***0.000***	0.956	*0.473*	0.935	*0.228*
	Veterinary Medicines	**0.675**	***0.000***	0.930	*0.254*	0.957	*0.417*
	Large Livestock	**0.612**	***0.000***	0.987	*0.826*	**0.827**	***0.001***
	Small Livestock	**0.708**	***0.000***	**0.829**	***0.014***	1.048	*0.473*
	Irrigation	1.038	*0.716*	**0.884**	***0.023***	**1.367**	***0.002***
Agricultural Practices	Cover Crop	**1.418**	***0.030***	**0.895**	***0.037***	1.136	*0.387*
	Mulch	**1.532**	***0.000***	**0.877**	***0.016***	**1.228**	***0.003***
	Crop Rotation	1.076	*0.303*	**0.901**	***0.060***	0.990	*0.862*
	Water Efficiency	**0.621**	***0.000***	**0.859**	***0.006***	1.000	*1.000*
Financial and Social	Agricultural Credit	**0.783**	***0.015***	**0.898**	***0.046***	0.940	*0.402*
	Cash Other Farm	**1.652**	***0.000***	**0.890**	***0.045***	1.022	*0.676*
	Cash Other Business	**0.818**	***0.002***	**0.864**	***0.008***	**1.136**	***0.023***
	Informal Loan	0.909	*0.168*	**0.906**	***0.075***	0.942	*0.312*
	Formal Loan	**0.701**	***0.000***	**0.909**	***0.079***	**0.837**	***0.019***
	Cash Gifts	1.027	*0.706*	**0.867**	***0.011***	**1.144**	***0.022***
	Group Participation	**0.722**	***0.000***	1.095	*0.141*	**0.723**	***0.000***
	Female Household	**1.474**	***0.000***	**0.891**	***0.033***	**1.140**	***0.082***

Full models with random effects and confidence intervals are reported in Supplementary Table 4. Statistically significant results (*p* < 0.10) are bold.

### Agricultural Input Factors

Overall we find across all households that most agricultural factors independent of rainfall anomaly (i.e. main effects) are significantly associated with decreased odds of food insecurity including fertilizer (0.797), pesticides (0.787), veterinary medicines (0.675), large livestock (0.612), and small livestock (0.708) (*p* > 0.01). We find no significant effect overall of the relationship between using manure or irrigation and food insecurity and find that use of certified seeds results in 1.223 greater odds of food insecurity (*p* > 0.01). Interaction effects demonstrate that there are not statistically significant differences with having certified seeds, fertilizers, pesticides, veterinary medicines, or small livestock in interaction with rainfall (Table 1). We find that there are statistically significant differences with manure use in interaction with rainfall anomaly, which correlates with greater odds of food insecurity (1.136, *p* < 0.05), while large livestock in context of rainfall anomaly correlates with reduced odds of food insecurity (0.827, *p* < 0.01).

**Figure 3:**
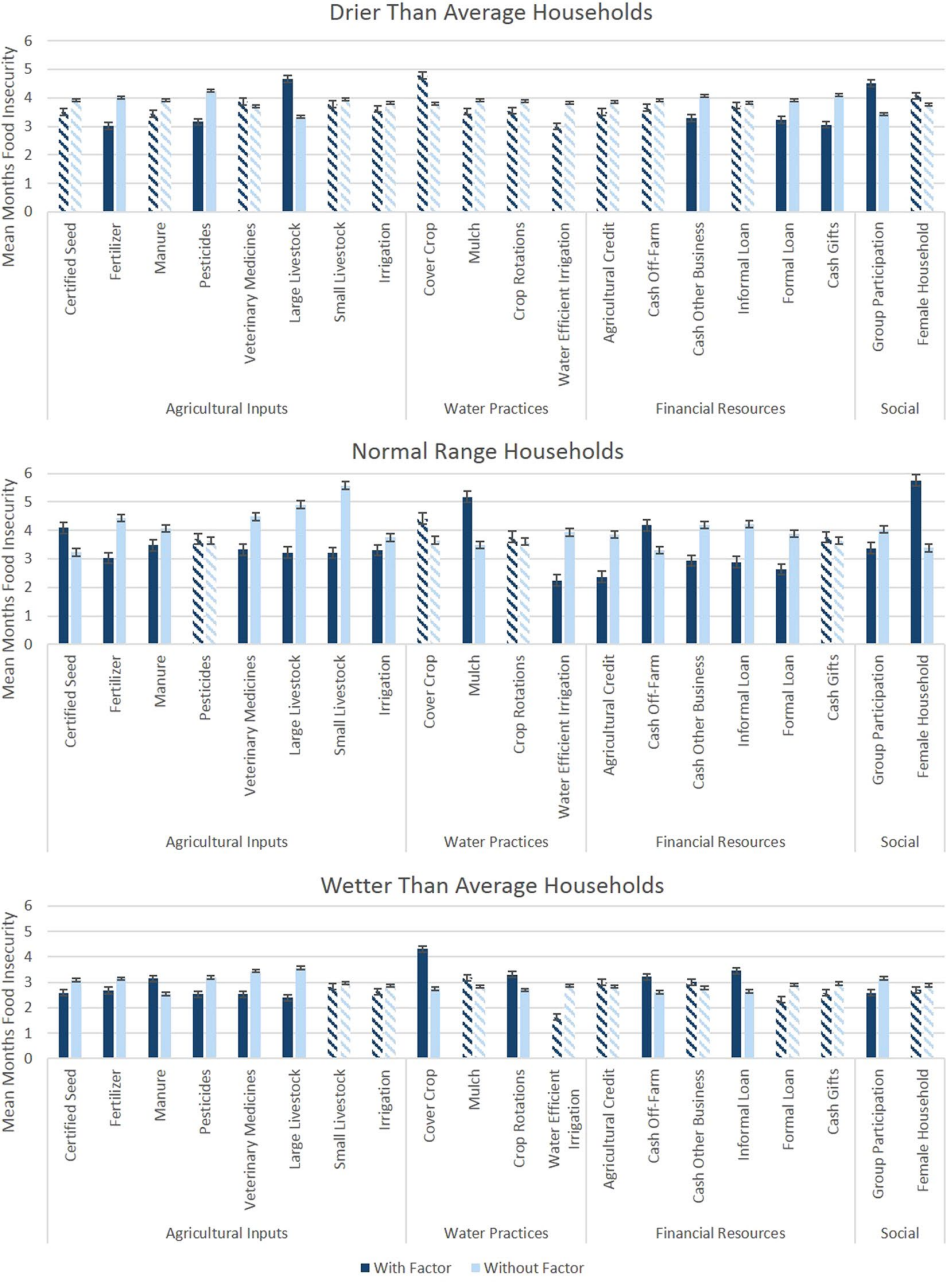
ANOVA results of mean months of food insecurity in the presence or absence of a given factors across the three household types. Solid bars represent statistically significant results as reported in the text, while dashed bars represent non-significant results. Statistical significance is between bars of a given factor (i.e. whether a household with or without the factor has statistically significant differences in mean months food insecurity). Standard errors are shown on top of bars.

Given that we are interested not only in the overall effect of these factors, but also their use in different household conditions of rainfall, we also explored the mean levels of food insecurity across different household types (Table 2, Fig. 3). We find that there is a statistically significant association with reduced odds of food insecurity across all household types with fertilizer use (*p* < 0.05). In addition, in normal range households, manure use, veterinary medicines, large livestock, and small livestock (*p* < 0.01) and irrigation (*p* < 0.10), are associated with lower mean months of food insecurity while use of certified seed is associated with higher average months of food insecurity (*p* < 0.000). In drier than average households, pesticide use results in lower average months of food insecurity (*p* < 0.000), while ownership of large livestock results in higher average months of food insecurity (*p* < 0.000). In wetter than average households, certified seed, fertilizer, pesticides, veterinary medicines, and large livestock are all associated with decreased months of average food insecurity (*p* < 0.05), while manure use is associated with higher average months of food insecurity (p < 0.01).

**Table 2:** Mean months food insecure by household type based on standard deviation of rainfall anomaly in the presence or absence of different factors.

		Drier Than Average		*p*	Normal Range		*p*	Wetter Than Average		*p*
		With Factor	Without Factor		With Factor	Without Factor		With Factor	Without Factor	
Agricultural Inputs	Certified Seed	3.5 (n = 115)	3.93 (n = 298)	0.161	4.09 (n = 467)	3.23 (n = 446)	0.000	2.58 (n = 292)	3.09 (n = 337)	0.023
	Fertilizer	3.02 (n = 84)	4.01 (n = 329)	0.004	3.03 (n = 494)	4.43 (n = 419)	0.000	2.68 (n = 395)	3.15 (n = 234)	0.044
	Manure	3.45 (n = 95)	3.92 (n = 318)	0.161	3.48 (n = 623)	4.07 (n = 430)	0.003	3.15 (n = 319)	2.55 (n = 310)	0.008
	Pesticides	3.16 (n = 164)	4.24 (n = 249)	0.000	3.7 (n = 499)	3.63 (n = 414)	0.699	2.53 (n = 323)	3.2 (n = 305)	0.003
	Veterinary Medicines	3.87 (n = 262)	3.71 (n = 151)	0.574	3.32 (n = 638)	4.48 (n = 275)	0.000	2.54 (n = 410)	3.45 (n = 218)	0.000
	Large Livestock	4.66 (n = 150)	3.33 (n = 263)	0.000	3.22 (n = 583)	4.9 (n = 271)	0.000	2.40 (n = 384)	3.57 (n = 245)	0.000
	Small Livestock	3.78 (n = 344)	3.94 (n = 69)	0.673	3.21 (n = 762)	5.57 (n = 54)	0.000	2.83 (n = 525)	2.97 (n = 103)	0.649
	Irrigation	3.61 (n = 18)	3.82 (n = 395)	0.760	3.30 (n = 174)	3.75 (n = 879)	0.057	2.63 (n = 32)	2.87 (n = 597)	0.635
Agricultural Practices	Cover Crop	4.78 (n = 9)	3.78 (n = 404)	0.301	4.41 (n = 47)	3.65 (n = 1,006)	0.358	4.32 (n = 40)	2.75 (n = 589)	0.001
	Mulch	3.52 (n = 105)	3.91 (n = 308)	0.227	5.17 (n = 96)	3.49 (n = 957)	0.000	3.18 (n = 51)	2.83 (n = 578)	0.395
	Crop Rotations	3.55 (n = 106)	3.90 (n = 307)	0.267	3.77 (n = 359)	3.6 (n = 694)	0.396	3.30 (n = 165)	2.70 (n = 464)	0.018
	Water Efficient Irrigation	3.00 (n = 6)	3.82 (n = 407)	0.479	2.25 (n = 139)	3.93 (n = 914)	0.000	1.63 (n = 8)	2.87 (n = 621)	0.209
Financial and Social	Agricultural Credit	3.51 (n = 59)	3.86 (n = 354)	0.376	2.37 (n = 111)	3.85 (n = 802)	0.000	3.00 (n = 58)	2.84 (n = 570)	0.680
	Cash Off-Farm	3.65 (n = 158)	3.91 (n = 255)	0.367	4.18 (n = 386)	3.30 (n = 527)	0.000	3.22 (n = 246)	2.62 (n = 383)	0.009
	Cash Other Business	3.29 (n = 132)	4.06 (n = 281)	0.010	2.94 (n = 376)	4.19 (n = 537)	0.000	3.01 (n = 190)	2.79 (n = 439)	0.358
	Informal Loan	3.73 (n = 135)	3.84 (n = 278)	0.698	2.89 (n = 375)	4.22 (n = 537)	0.000	3.46 (n = 150)	2.65 (n = 479)	0.002
	Formal Loan	3.23 (n = 62)	3.91 (n = 351)	0.077	2.63 (n = 157)	3.89 (n = 755)	0.000	2.31 (n = 59)	2.91 (n = 570)	0.113
	Cash Gifts	3.06 (n = 111)	4.09 (n = 302)	0.001	3.76 (n = 316)	3.63 (n = 597)	0.500	2.59 (n = 169)	2.95 (n = 460)	0.146
	Group Participation	4.52 (n = 143)	3.44 (n = 270)	0.000	3.37 (n = 490)	4.03 (n = 423)	0.000	2.58 (n = 331)	3.16 (n = 298)	0.011
	Female Household	4.07 (n = 75)	3.75 (n = 337)	0.384	5.75 (n = 112)	3.38 (n=801)	0.000	2.70 (n=69)	2.88 (n=560)	0.614

Total number of smallholder farmers in each category is provided in parentheses.

### Agricultural Practices

Many of the agricultural practices we explored had very low adoption rates, particularly in drier than average and wetter than average conditions. This should be considered when interpreting these results (e.g. cover crop adoption). In exploring agricultural practices with water-related potential outcomes, we find that the use of cover crops (1.148) and mulch (1.532) is correlated with higher odds of food insecurity (*p* < 0.01). Conversely, water use efficiency is associated with decreased odds of food insecurity (0.621, *p* < 0.000). We find that only mulch has a statistically significant interaction effect, with greater odds of food insecurity (1.228, *p* < 0.01) as rainfall anomalies trend towards normal or wet. Our ANOVA analysis (Fig. 3) corroborates this by demonstrating that mulch is associated with lower rates of food insecurity in drier than average households (though not significantly, *p* = 0.227), while it is correlated with higher rates of food insecurity in normal range households (*p* < 0.000). Both cover crops and crop rotations are associated with higher rates of food insecurity in wetter than average households (*p* < 0.05), while water efficient irrigation is significantly associated with reduced average food insecurity in normal range households (*p* < 0.000).

### Financial/Social Factors

We find that three financial factors are positively associated with decreased odds of food insecurity overall, including agricultural credit (0.783, *p* < 0.05), cash from other businesses (0.818, *p* < 0.01), and formal loans (0.701, *p* < 0.000). Cash from other farm work (typical of landless laborers) is correlated with a 1.652 greater odds of food insecurity (*p* < 0.000) and we find no significant effect of informal loans or cash gifts overall. We find that group participation is positively correlated with decreased odds of food insecurity (0.722, *p* < 0.000) while female-headed households are correlated with a 1.474 increased odds of food insecurity (*p* < 0.000). We find statistically significant interaction effects for increased food insecurity odds with cash from other businesses (1.136, *p* < 0.05) and cash gifts (1.144, *p* < 0.05), indicating that as rainfall anomalies become more normal or wet, (i.e. households have greater rainfall) having these factors are no longer associated with reduced food insecurity (see Supplementary Materials Fig. 2–4 for visual relationship). Conversely, we find decreased food insecurity odds for interaction effects with formal loans (0.837*, p* < 0.05), and group participation (0.723, *p* < 0.000), indicating that as rainfall anomalies become more normal or wet, these factors continue to decrease food insecurity.

ANOVA results (Fig. 3) indicate statistically significant lower average months of food insecurity in normal range households for those with agricultural credit, cash from other businesses, informal loans, formal loans, and group participation (*p* < 0.000), while average months of food insecurity are higher in households with off-farm cash income (e.g. income earned working on other farms, often associated with landless laborers) and female-headed households. Cash from other businesses and cash gifts (*p* < 0.01) and formal loans (*p* < 0.10) are significantly associated with decreased months of food insecurity in drier than average households, while group participation is significantly associated with increased average months of food insecurity (p < 0.000). Informal loans and group participation are also significantly associated with decreased number of months of food insecurity in wetter than average households (*p* < 0.01).

## Discussion

Examining relationships between food insecurity and varying agricultural, financial, and social factors reveals several important outcomes when considering rainfall anomalies across multiple countries and sites. First, we find clear evidence that rainfall anomalies are correlated with food insecurity. Drier than average households had 3.81 months mean food insecurity while normal range households had 3.67 mean months food insecurity and wetter than average households had 2.86 months mean food insecurity. Given these regions predominantly rely on rain-fed agriculture^31,32^, these results could suggest a variety of impacts as lack of rainfall may manifest in reduced income from agriculture due to the damage of crops from lack of rainfall, resulting in a contraction of the agriculture-based economy^33^.

These results are consistent with research that has largely focused on individual countries, finding for example that smallholders in Ethiopia were less food insecure in wetter and less variable rainfall regions^34^. This evidence suggests that infrastructure investments for irrigation in appropriate places may provide a potential climate adaptation strategy for rainfall variable regions that are predicted to become drier, though such strategies must carefully consider the benefits and challenges of irrigation expansion including the potential for farmers to shift to cash crops^35,36^.

Second our results suggest that among two otherwise equal households, those having certain agricultural inputs (fertilizers, pesticides, veterinary medicines, large livestock, small livestock) agricultural practices (water efficient irrigation), financial factors (agricultural credit, cash from other businesses, formal loans) and group participation are all correlated with reduced odds of food insecurity outside the context of rainfall anomalies (Table 1). Conversely, use of certified seeds, cover crops, mulch, cash from work on another farm, and female-headed households are all correlated with higher odds of food insecurity. These results demonstrate that overall, these development interventions could provide smallholder farmer households with strategies to reduce food insecurity without consideration for rainfall.

Livestock, for example, are sought out by smallholder farmers and often act as key self-insurance mechanisms^37^, and have been associated with increased productivity^38^ and income-levels at the farm level^39^, which could also explain its relationship to food insecurity. Conversely, cash from work on another farm is correlated with increased odds of food insecurity, which may be sought for particularly vulnerable households or homes without adequate land base^40,41^. This cash income from working on another farm may also be associated with migration for work, and is particularly vulnerable to changes in demand for agricultural laborers due to drought^42^. For example, Murali and Afifi^43^ found seasonal migration in India as the most frequent coping strategy for rice farmers in the region to deal with rainfall variability, while Bhatta *et al*.^29^ found short-term migration the most likely for farmers in India and Bangladesh. Warner and Afifi (2014)^25^ examined how migration associated with rainfall variability is an important strategy for safeguarding food security across eight countries and others have noted it’s a critical coping strategy in dry seasons in Ghana^44^.

Third, however, when considering households in a rainfall context, we find much more nuanced results, in which interventions and their correlated food insecurity vary significantly by rainfall anomalies. In drier than average households, the most food insecure, we find that agricultural inputs and agricultural practices are rarely associated with decreased odds of food insecurity. Only the use of fertilizers and pesticides is associated with decreased odds of food insecurity. Conversely, we find that more financial factors are likely to be correlated with decreased odds of food insecurity through cash from other businesses, formal loans and cash gifts. This is consistent with recent research suggesting that for especially marginal households, production oriented intensification practices may not provide adequate responses to food insecurity^45^ and that a focus on greater access to market and financial resources may be a better strategy for maintaining food consumption in the face of reduced household food availability and access^46,47^.

For normal range households, a far greater number of strategies across agricultural inputs, practices and financial and social factors are correlated with reduced odds of food insecurity, while for wetter than average households, it is agricultural inputs that are most frequently correlated with reduced odds of food insecurity, with financial factors being entirely insignificant. This suggests that water is fundamentally a limiting factor for many of these regions; when it is abundant, the use of agricultural inputs noticeably improves food security; however, with a lack of rainfall, agricultural inputs may not be a viable strategy. As suggested by Niles *et al*.^48^, limiting factors at the farm level could be key drivers of farmer behaviors for adaptation, and Wood *et al*.^27^ find evidence that climatic factors are causing smallholder farmers to shift to different practices. This work provides context for the conditions in which such shifts may have positive food security benefits.

Further, we find several cases of factors that have varying effects across rainfall anomalies. For example, manure use, irrigation, mulching, cash from other businesses, and cash gifts have significant interaction effects in combination with rainfall, indicating that there are significantly different results depending on rainfall. ANOVA results suggests that these factors trend towards improved food security in drier than average conditions, but do not necessarily result in the same effect in normal and wetter than average years (see visual representations in Supplementary Figures 2–4). For example, while cash from other businesses results in fewer months of food insecurity in drier than average and normal range households, it is not statistically significant in wetter than average years. Cash remittances are statistically significant for reducing food insecurity in drier than average households, but are not significant in other households^49^. It is likely that the additional cash income is critical in times of reduced rainfall, when crop yields could be compromised, resulting in reduced farm income, affecting both food access and availability^15^. Thus, alternative sources of income can help overcome production losses. Conversely, large livestock and group participation result in greater odds of food insecurity in drier than average households, but significantly reduced odds of food insecurity in normal range and wetter than average households. It may be that since livestock are often critical sources of insurance for long-term risk^28^, smallholders continue to invest in their livestock despite lack of rainfall, diverting potential food or fuel as well as cash income towards livestock rather than maintaining household consumption.

Our results also highlight the critical need for continued focus on women and female-headed households, as these households were significantly more likely to be food insecure across all households regardless of percent change in rainfall. The literature suggests that the relationship of gender and food security is complex since women often produce a significant portion of agricultural production in the developing world, but lack access to adequate resources including credit and inputs or highly productive land, which can typically explain the gap in productivity^28,50,51^.

Interventions that are “one size fits all” and which could be expected to work in all places and all conditions are nearly non-existent in this dataset - with the exception of fertilizer, which we find to consistently, across all rainfall anomalies, result in greater odds of reduced food insecurity. Fertilizer has shown this effect previously but in better rainfall conditions in Malawi, where fertilizer subsidies increased maize production^52,53^. Given the potential for fertilizer to increase yields, particularly in Sub-Saharan Africa, its use can also increase crop residues, which can provide livestock feed, potential fuel, and soil conservation benefits^34^, all of which may be related to the negative correlation with food insecurity we find here.

There are very few silver bullets- development interventions that are available and used could have different outcomes for food insecurity depending on the rainfall and climatic conditions of a given place. For significantly drier than average years, our results indicate that financial interventions may be more important for food security- access to cash from either remittances, other businesses and formal loans can help households maintain food consumption and productive assets during a drought and drier years. Financial institutions have recently renewed programs that work to find ways to provide smallholder farmers with credit in low income countries, but the need remains largely unmet^54^. Additional efforts focused on providing diverse financial services may offer further benefits beyond a singular focus on agricultural inputs. New digital and mobile technologies offer great potential to target smallholders who may have traditionally been unable to assess financial resources previously and should continue to be an important development objective^55,56^. Conversely, when rainfall is abundant, as it was in wetter than average households, it was the use of agricultural inputs, that most significantly correlated with reduced odds of food insecurity.

## Conclusion

Increasing livelihood outcomes for smallholder farmers is a critical goal for ensuring future food security, a goal that will require multiple strategies and varying scales for success. Taken together in the context of a changing climate, these results demonstrate that improving smallholder farmer livelihoods will be further complicated by increased rainfall variability and climate changes as drier than average conditions are strongly correlated with increased food insecurity. While our evidence suggests that a number of factors affect the odds of food insecurity, agricultural, financial and social factors are significantly correlated with decreasing food insecurities in different contexts, importantly highlighting that one size fits all solutions are not likely or viable in varying climatic conditions.

We find that more financial factors - cash from other businesses, formal loans and cash gifts- along with pesticides and fertilizers have the largest correlation with reduced odds of food insecurity in households facing drier than average conditions, which are also more likely to be food insecure. Regardless of rainfall change, fertilizers, pesticides, veterinary medicines, large and small livestock, water use efficiency, agricultural credit, and cash from other businesses, formal loans, and group participation are all positively correlated with food security while female-headed households cover crops, mulching, and cash from other farms are correlated with increased odds of food insecurity.

Given that most developing world smallholder farmers still rely on rain-fed agriculture, and that rainfall variability is expected to increase in the coming decades, our work demonstrates that expansion of both agricultural inputs and practices, but especially financial services, may provide notable benefits for food security and likely many other aspects of well-being. We believe that a greater focus on evaluating the impact of non-farm production oriented strategies, including financial strategies, to improve food security should be considered in further research. We acknowledge a limitation of this work- it is based on data which provides only a single point in time. Having repeat visits to the same households across multiple years would enable a greater ability to look at households over time, perhaps across varying kinds of rainfall years. Thus, it is imperative that longer-term datasets be collected among smallholder farmers to enable a better understanding of the impact of climatic variables on food security interventions. A focus on collecting data beyond single time points can provide improved understanding of the role of multiple interventions on food security and other livelihood strategies, which are critical for climate change adaptation and improved food security in the future.

## Methods

The CCAFS program collected data from 15 sites during their baseline survey assessment from December 2010 through 2012. These sites were not chosen at random, rather they were selected for their focus on smallholder agricultural communities that are typically low-income. Here we omit data from Haryana, India, per the advice of CCAFS, given the inaccuracy of their measurement on food security^57^. In each location, approximately 140 households were randomly selected from a 10 by 10 km^2^ sampling frame or a 30 by 30 km^2^ area in places of low population density. In total, this dataset contains 1,955 household surveys from 101 villages and 14 community sites. Additional information regarding the data collection can be found in Förch *et al*.^58^.

The Climate Hazards Group InfraRed Precipitation with Station (CHIRPS), a 30+ year quasi-global high-resolution rainfall dataset, was used to estimate agriculturally relevant rainfall anomalies. The data incorporates 0.05 degree resolution satellite imagery with *in-situ* station data to create a gridded time series for identification of trends and rainfall anomalies that have socioeconomic importance (13). CCAFS baseline survey data included the random offset GPS coordinates for each household survey location, which were matched with CHIRPS data from the 30 years prior to the date of the survey at the household level (ranging from 1981 to 2012 depending on the survey date at the site). GPS coordinates for households were jittered 0.002 in longitude and 0.001 in latitude, which should not significantly influence these results. Using this data we determined the three wettest cumulative months per year (growing season) (Supplementary Table 1). These three months were summed annually and then an annual average sum was calculated over a 30-year time period, a generally recognized period to demonstrate a long-term climatological average^59^, providing the average annual sum of the three wettest cumulative months at each household location. We recognize that temperature is also an important variable and is closely related to precipitation in tropical convective systems^60^, but it is excluded from this analysis to maintain the spatial and temporal specificity in the analysis enabled by the long time series, 5 km resolution CHIRPS data.

Since our dependent variable of interest is food insecurity within the last twelve months (see further details below), we assessed how the rainfall in the year prior to the survey (previous 12 months) was different than the 30 year climatological mean. We calculated standardized anomalies for each household, by dividing anomalies by the climatological standard deviation, which enables a greater understanding of the magnitude of the anomaly since influences of dispersion have been removed^59^. These standardized anomalies ranged from −2.656 standard deviations below the 30 year mean for a given household to 1.923 standard deviations above the 30 year mean for a given household (Supplementary Figure 2). Thus, we compiled three types of households within the rainfall anomaly: 1) those that were more than one standard deviation or more below the 30 year mean, which we call “Drier Than Average” households for the year prior to the survey (n = 413); 2) those that were within one standard deviation above or below the mean, which we call “Normal Range” households (n = 1,053); 3) those that were above one standard deviation or more above the mean, which we call “Wetter Than Average” households (n = 629). We also report average growing season temperatures in the Supplementary Materials, calculated using Climate Hazards Group InfraRed Temperature with Station (CHIRTS), a 30+ year quasi-global high-resolution temperature dataset.

These standardized anomalies were coupled with CCAFS baseline survey data about household access to various agricultural inputs (certified seeds, fertilizer, pesticides, veterinary medicines, large and small livestock, irrigation), agroecological practices (manure use, cover crops, mulching, crop rotations, water efficiency), and financial and social factors (agricultural credit, cash from work on other farms, cash from other businesses, informal loans, formal loans, cash gifts, group participation and female-headed households) to assess their relationship to food security. Site means (n = 14) for all variables are listed in Supplementary Table 2. Supplementary Table 3 lists the model variables and survey questions. In addition, we provide the entire CCAFS household survey for reference in the supplementary materials.

Given our dependent variable has 12 possible months of food insecurity, we developed hierarchical mixed random effects logit models with binomial responses across 12 trials, one for each month^61^ to explore the relationship of standardized rainfall anomalies in interaction with different household factors across the three household types- drier than average, normal range, and wetter than average. This approach allows for a household specific adjustment to the log-odds baseline of each monthly outcome. This approach is appropriate when data is not normally distributed because it enables better prediction of outcomes at the “tail” of the data- in this context, households that are very food insecure^61^, a factor we do not want to underestimate. We include random effects at the household and village level, which enable the comparison of variables in the context of otherwise equal households. These varying random effects can control for the unobserved differences in food security associated with clustered data^19^. These types of models control for spatial variability and thus can account for any unseen effects that might influence food insecurity as a result of geography, governance, maturity of the local agricultural or financial markets, or other spatial relationships which might confound our analysis. We ran a total of 20 different multi-level random effects models to assess the relationship of each factor and standardized rainfall anomaly independently and its interaction with food insecurity, reporting the main effects and interaction effects, which can be interpreted as the log-odds of an outcome.

However, given that households that are drier than average are more food insecure than those within the normal range and wetter than average, we also utilized an analysis of variance (ANOVA) to explore significant differences in food insecurity based on the three household types. We used a two-way factorial ANOVA to determine the relationship of the presence or absence of a factor in drier, normal range, or wetter than average households on food insecurity^62^. These analyses thus enable greater understanding about how a given factor in different rainfall conditions is related to average levels of food insecurity. We report the mean months of food insecurity across these household types, as well as the p-value and standard errors. Food security was measured on a monthly basis such that respondents were asked to indicate which months they had a shortage of food or struggled to feed their family (full questions in the analysis and questionnaires are included in Supplementary Materials).

## Electronic supplementary material

Supplementary Materials
