# Supplementary material for: A multi-country assessment of factors related to smallholder food security in varying rainfall conditions
Source: Sci Rep. 2017 Nov 24;7:16277. doi: 10.1038/s41598-017-16282-9 (PMC5701123; doi:10.1038/s41598-017-16282-9)
Supplement: Supplementary file 1 — Supplementary Materials [file 41598_2017_16282_MOESM1_ESM.docx]

**Title**: **A multi-country assessment of factors related to smallholder food security in varying rainfall conditions**

Meredith T. Niles and Molly E. Brown

**Supplementary Materials**

Figure 1. Standardized anomalies of rainfall as compared to 30 year mean. Households that were drier than average (n=413) were on average -1.74 standard deviations below the mean (standard deviation= 0.558) with a range of -2.656 to -1.052. Households that were within the normal range (n=1053) were on average 0.007 standard deviations above the mean (standard deviation= 0.587) with a range of -0.973 to 0.999). Households that were wetter than average (n=629) were on average 1.30 standard deviations above the mean (standard deviation=0.262) with a range of 1.008 to 1.923).


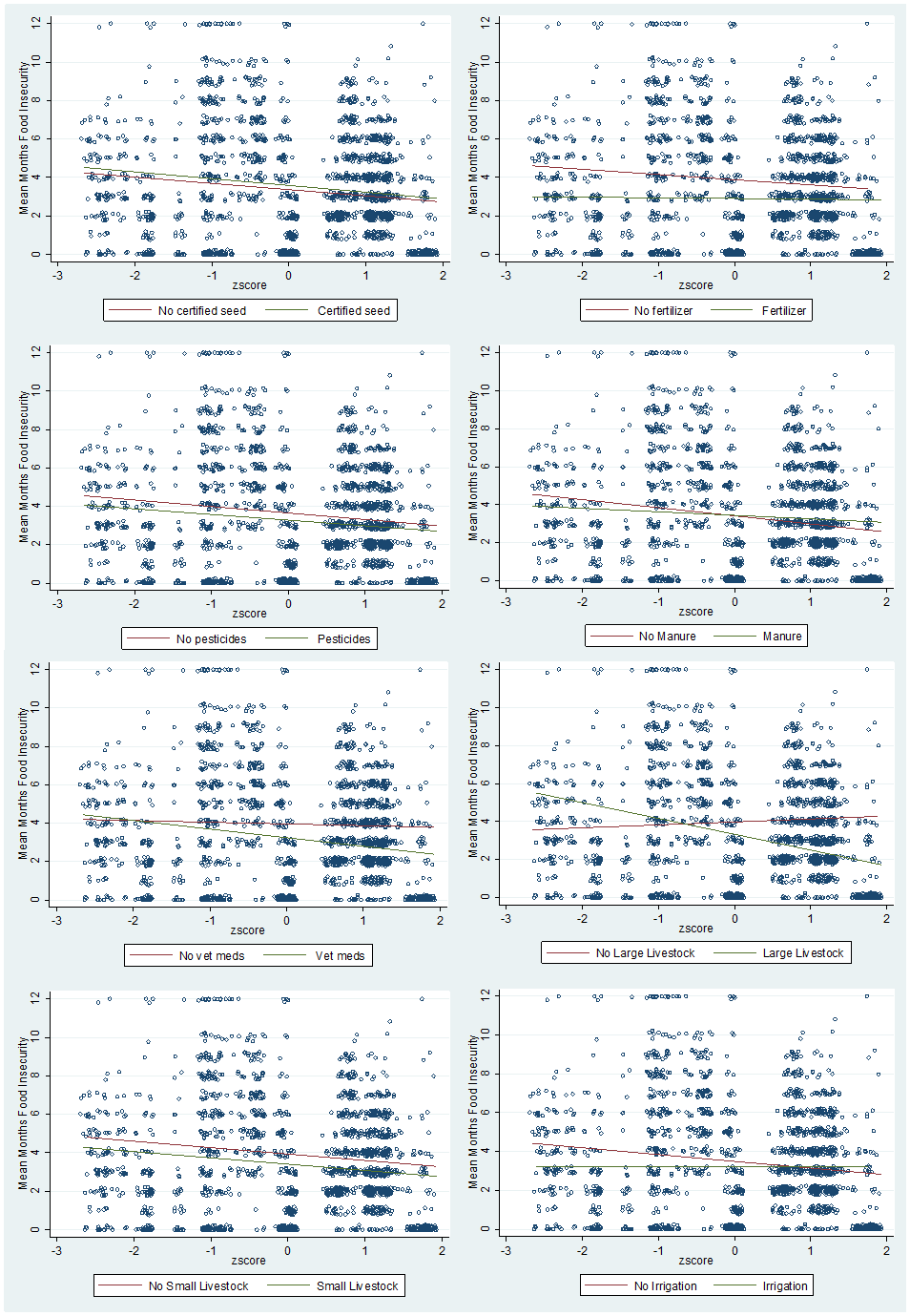


**Figure 2.** Graphical results of multi-level random effects models across the eight agricultural input factors. Graphs show the scatter plot of all households’ standardized rainfall anomaly plotted against food insecurity data, with the slope coefficients from individual models of the presence and absence of a given factor. Manure and large livestock have statistically significant different slopes depending on rainfall anomaly.


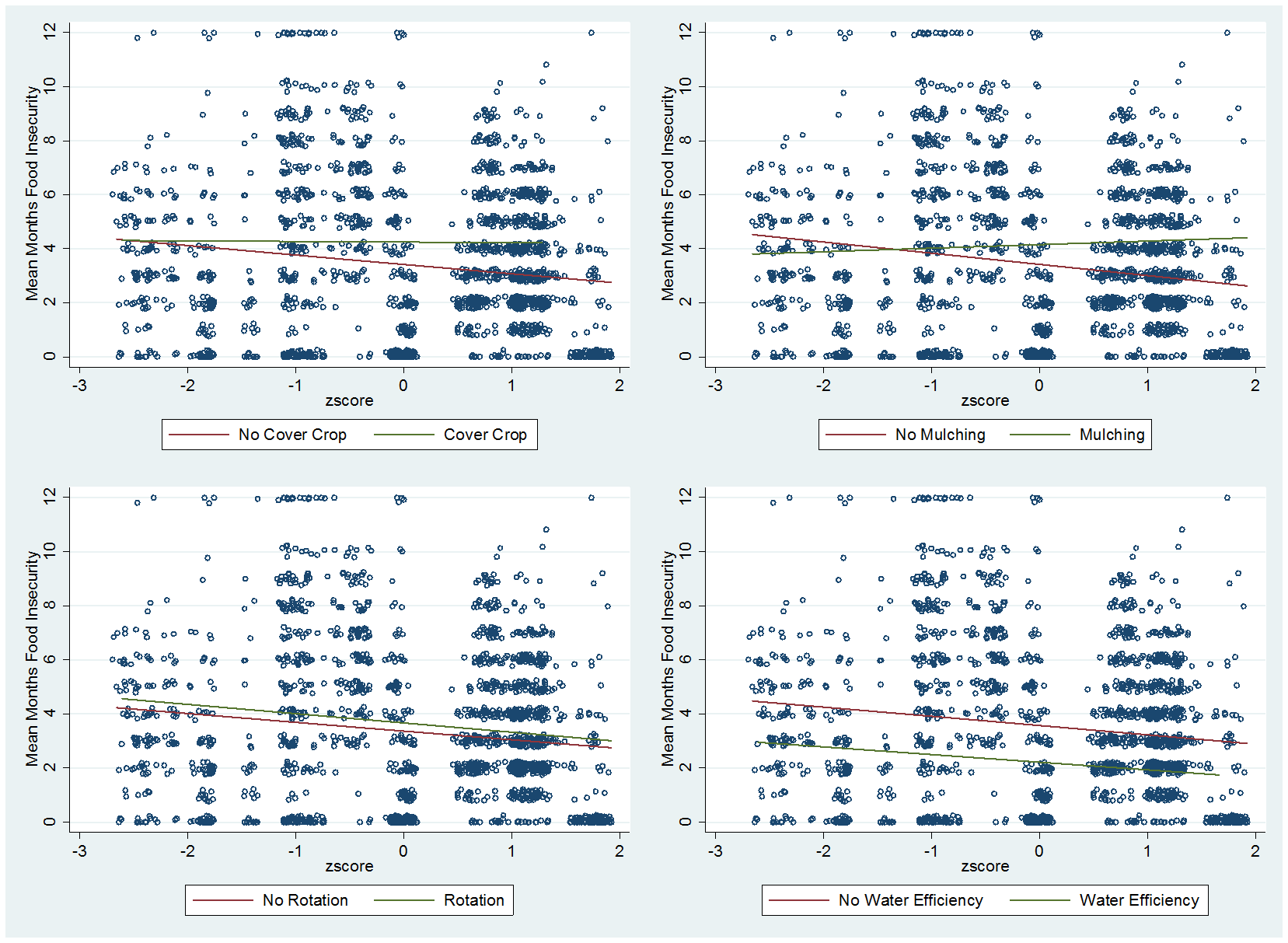


**Figure 3.** Graphical results from multi-level models, indicating that water efficiency practices are correlated with decreased food insecurity, while cover crops and mulching are overall associated with higher levels of food insecurity.


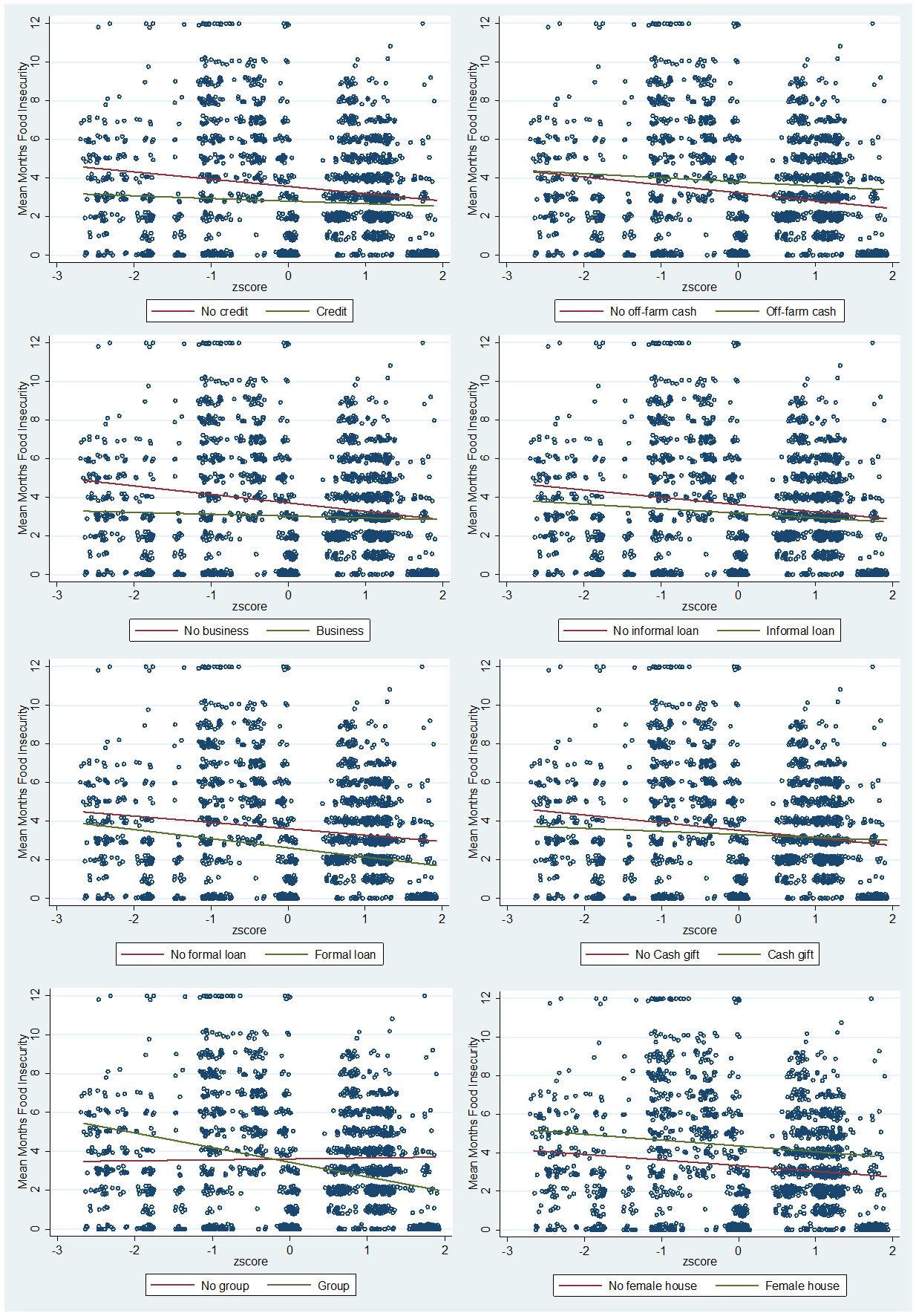


**Figure 4.** Graphical results of multi-level random effects models across the eight financial and social factors. Results indicate that agricultural credit, cash from other businesses, formal loans, and group participation are correlated with reduced food insecurity, while cash from work on other farms and female-headed households are correlated with higher rates of food insecurity overall.

| \| **Supplementary Table 1.** Growing season months used for three wettest consecutive months for rainfall and temperature anomaly calculation \| \| \| --- \| --- \| \| **Site Name** \| **Three Wettest Months** \| \| Bangladesh \| June, July, August \| \| Burkina Faso \| July, August, September \| \| Ethiopia \| September, October, November \| \| Ghana \| July, August, September \| \| India (Karnal) \| July, August, September \| \| Kenya (Katuk) \| July, August, September \| \| Kenya (Wote) \| October, November, December \| \| Mali \| July, August, September \| \| Nepal \| July, August, September \| \| Niger \| July, August, September \| \| Senegal \| July, August, September \| \| Tanzania \| March, April, May \| \| Uganda (Hoima) \| August, September, October \| \| Uganda (Rakai) \| March, April, May \|     **Supplementary Table 2.** Site and global mean of model variables | | | | | | | | | | | | | | | | |
| --- | --- | --- | --- | --- | --- | --- | --- | --- | --- | --- | --- | --- | --- | --- | --- | --- | --- | --- | --- | --- | --- | --- | --- | --- | --- | --- | --- | --- | --- | --- | --- | --- | --- | --- | --- | --- | --- | --- | --- | --- | --- | --- | --- | --- | --- | --- | --- | --- |
| Variable | Description | Bang  ladesh | Burkina Faso | Ethiopia | Ghana | India Karnal | Kenya Katuk | Kenya Wote | Mali | Nepal | Niger | Senegal | Tanzania | Uganda Hoima | Uganda Rakai | Global |
| Food Security | Months food insecure in the previous year | 3.4 | 3.8 | 6.6 | 4.4 | 2.4 | 1.9 | 6.1 | 0.9 | 1.0 | 3.7 | 2.4 | 5.4 | 2.5 | 3.8 | 3.4 |
| Certified Seed | Purchased certified seed in previous 12 months (%) | 20% | 54% | 28% | 24% | 91% | 64% | 96% | 18% | 61% | 31% | 18% | 70% | 30% | 48% | 48% |
| Fertilizer | Purchased fertilizer in previous 12 months (%) | 64% | 73% | 0% | 56% | 91% | 20% | 6% | 33% | 100% | 66% | 72% | 81% | 17% | 20% | 52% |
| Manure | Started using manure/compost in the last 10 years | 4% | 74% | 0% | 91% | 77% | 33% | 91% | 55% | 31% | 46% | 93% | 83% | 17% | 49% | 49% |
| Pesticides | Purchased pesticides in previous 12 months (%) | 64% | 37% | 7% | 63% | 77% | 23% | 84% | 13% | 87% | 41% | 54% | 66% | 31% | 60% | 53% |
| Vet. Meds | Purchased vet. meds in previous 12 months (%) | 64% | 89% | 84% | 38% | 51% | 79% | 69% | 87% | 71% | 51% | 89% | 59% | 53% | 59% | 68% |
| Large Livestock | Produced large livestock in past 12 months (%) | 42% | 59% | 19% | 73% | 19% | 21% | 71% | 66% | 66% | 86% | 44% | 58% | 81% | 91% | 59% |
| Small Livestock | Produced small livestock in past 12 months (%) | 91% | 94% | 94% | 99% | 37% | 87% | 91% | 96% | 74% | 71% | 96% | 83% | 81% | 81% | 84% |
| Irrigation | Started irrigating in the last 10 years | 4% | 25% | 0% | 0% | 38% | 8% | 19% | 0% | 1% | 0% | 9% | 44% | 4% | 9% | 11% |
| Cover Crop | Started using cover crops in the last 10 years | 0% | 15% | 2% | 21% | 6% | 1% | 4% | 1% | 0% | 1% | 0% | 12% | 2% | 4% | 5% |
| Mulch | Started using mulch in the last 10 years | 1% | 24% | 0% | 9% | 3% | 3% | 22% | 1% | 5% | 3% | 0% | 36% | 19% | 56% | 12% |
| Crop Rotation | Started using crop rotation in the last 10 years | 14% | 37% | 21% | 70% | 12% | 12% | 69% | 8% | 16% | 14% | 93% | 28% | 34% | 24% | 30% |
| Water Efficiency | Introduced improved irrigation (water efficiency) in the last 10 years | 14% | 4% | 0% | 0% | 79% | 1% | 1% | 0% | 1% | 0% | 0% | 7% | 1% | 2% | 7% |
| Agricultural Credit | Utilized credit in the previous 12 months (%) | 6% | 8% | 1% | 14% | 16% | 2% | 2% | 25% | 4% | 22% | 12% | 9% | 16% | 26% | 14% |
| Cash Other Farm | Cash from other farm job in past 12 months (%) | 56% | 24% | 4% | 61% | 35% | 45% | 53% | 33% | 26% | 44% | 47% | 54% | 41% | 41% | 38% |
| Cash Other Business | Cash from non-farm business (%) | 35% | 40% | 19% | 52% | 16% | 45% | 29% | 65% | 11% | 15% | 64% | 34% | 34% | 40% | 35% |
| Informal Loan | Informal cash loan (%) | 74% | 37% | 9% | 31% | 36% | 4% | 36% | 56% | 7% | 48% | 62% | 14% | 31% | 28% | 34% |
| Formal Loan | Formal cash loan (%) | 30% | 11% | 9% | 9% | 11% | 6% | 9% | 38% | 14% | 4% | 23% | 2% | 16% | 16% | 14% |
| Cash Gift | Cash from remittances/gifts in past 12 months (%) | 21% | 29% | 11% | 38% | 47% | 45% | 42% | 20% | 27% | 2% | 40% | 50% | 37% | 37% | 30% |
| Group Participation | Membership in any community group (%) | 12% | 89% | 36% | 49% | 26% | 60% | 66% | 49% | 38% | 64% | 5% | 49% | 87% | 24% | 47% |
| Female House | Female-headed households (%) | 1% | 5% | 28% | 6% | 1% | 36% | 33% | 1% | 2% | 35% | 3% | 22% | 22% | 19% | 12% |
| Standardized Rainfall Anomaly | Standard Deviation of Rainfall change in previous year from 30 year average | -0.95 | 1.15 | -0.97 | 1.03 | -0.06 | 1.09 | -0.42 | 0.05 | 1.75 | 1.29 | 0.63 | 0.78 | -1.74 | -2.40 | 0.05 |
| Average Temperature (C) | Average growing season temperature | 32.36 | 33.38 | 25.15 | 30.77 | 33.82 | 28.42 | 29.19 | 32.22 | 34.27 | 33.95 | 33.13 | 24.60 | 26.88 | 26.30 | 30.32 |

| **Supplementary Table 3.** Model Variables, Questions and Scale. | | | |
| --- | --- | --- | --- |
| **Variable Type** | **Variable** | **Question or Context** | **Scale** |
| Agricultural Inputs | Certified Seed | In the last 12 months did you use any purchased certified/improved seed? | Yes=1, No=0 |
|  | Fertilizer | In the last 12 months did you use any purchase, inorganic/mineral fertilizer? | Yes=1, No=0 |
|  | Manure | Started using manure/compost in the last 10 years | Yes=1, No=0 |
|  | Pesticides | In the last 12 months did you purchase any pesticides/herbicides? | Yes=1, No=0 |
|  | Vet Meds | In the last 12 months did you purchase any veterinary medicines? | Yes=1, No=0 |
|  | Large Livestock | Production of any large livestock | Yes=1, No=0 |
|  | Small Livestock | Production of any small livestock | Yes=1, No=0 |
|  | Irrigation | Started irrigating in the last 10 years | Yes, =1, No=0 |
| Agricultural Practices | Cover Crops | Introduced cover cropping in the last 10 years | Yes, =1, No=0 |
|  | Mulch | Introduced mulching in the last 10 years | Yes, =1, No=0 |
|  | Crop Rotation | Introduced crop rotation in the last 10 years | Yes, =1, No=0 |
|  | Water Efficiency | Introduced water efficiency in the last 10 years | Yes, =1, No=0 |
| Financial/ Social Capital | Agricultural Credit | In the last 12 months did you get any credit for agricultural activities? | Yes=1, No=0 |
|  | Cash Other farm | In the last 12 months did any cash come into the household through employment on someone else's farm? | Yes=1, No=0 |
|  | Cash Business | In the last 12 months did any cash come into the household through business (other than farm products?) | Yes=1, No=0 |
|  | Informal Loan | During the last 12 months did any cash come to the household through loan/credit from an informal source (moneylender, relative, etc.) | Yes=1, No=0 |
|  | Formal Loan | During the last 12 months did any cash come to the household through loan/credit from a bank or other formal institution (microfinance, projects/programs, registered group) | Yes=1, No=0 |
|  | Cash Gifts | In the last 12 months did any cash come into the household through remittances or gifts? | Yes=1, No=0 |
|  | Group | Membership among any household member in a community group | Yes=1, No=0 |
|  | Female-headed | Any female-headed household for any reason | Yes=1, No=0 |
| Dependent | Food Security | Which months, if any, do you tend to find you do not have enough food to eat for your family | Scale 0-12 |
| Rainfall | Standardized Rainfall Anomaly | Standardized rainfall anomaly of the year prior to the survey compared to the 30 year climatological average for a given household location | Continuous |

| **Supplementary Table 4.** Model Odds Ratios in Interaction with Rainfall Change with Full Random Effects. Statistically significant results (*p* <0.10) are highlighted. | | | | | | | | | | | | | |
| --- | --- | --- | --- | --- | --- | --- | --- | --- | --- | --- | --- | --- | --- |
| Factors | Main Factor Effect | *p=* | Confidence Interval | Rainfall Effect (Absence of Factor) | Confidence Interval | *p=* | Interaction Effect (Factor with Rainfall) | Confidence Interval | *p=* | Constant | *p=* | Village Random Effect | Household Random Effect |
| Certified Seed | 1.223 | *0.004* | 1.066-1.403 | 0.896 | 0.796-1.001 | *0.067* | 1.002 | 0.900-1.116 | *0.969* | 0.333 | *0.000* | 0.472 | 1.134 |
| Fertilizer | 0.797 | *0.004* | 0.684-0.930 | 0.935 | 0.824-1.062 | *0.302* | 0.995 | 0.879-1.127 | *0.940* | 0.399 | *0.000* | 0.440 | 1.147 |
| Pesticides | 0.787 | *0.000* | 0.689-0.900 | 0.956 | 0.843-1.082 | *0.473* | 0.935 | 0.838-1.043 | *0.228* | 0.407 | *0.000* | 0.481 | 1.136 |
| Veterinary Medicines | 0.675 | *0.000* | 0.592-0.770 | 0.930 | 0.821-1.053 | *0.254* | 0.957 | 0.860-1.064 | *0.417* | -0.753 | *0.000* | 0.464 | 1.112 |
| Manure | 1.071 | *0.365* | 0.924-1.241 | 0.855 | 0.762-0.959 | *0.008* | 1.136 | 1.012-1.275 | *0.031* | 0.343 | *0.000* | 0.462 | 1.147 |
| Large Livestock | 0.612 | *0.000* | 0.535-0.701 | -0.134 | 0.875-1.112 | *0.826* | 0.827 | 0.738-0.928 | *0.001* | 0.490 | *0.000* | 0.448 | 0.975 |
| Small Livestock | 0.708 | *0.000* | 0.590-0.851 | 0.829 | 0.714-0.962 | *0.014* | 1.048 | 0.922-1.191 | *0.473* | 0.497 | *0.000* | 0.473 | 1.015 |
| Irrigation | 1.038 | *0.716* | 0.849-1.269 | 0.884 | 0.795-0.983 | *0.023* | 1.367 | 1.119-1.670 | *0.002* | 0.360 | *0.000* | 0.474 | 1.140 |
| Cover Crop | 1.418 | *0.030* | 1.035-1.944 | 0.895 | 0.806-0.993 | *0.037* | 1.136 | 0.851-1.516 | *0.387* | 0.355 | *0.000* | 0.458 | 1.142 |
| Mulch | 1.532 | *0.000* | 1.261-1.861 | 0.877 | 0.788-0.976 | *0.016* | 1.228 | 1.072-1.406 | *0.003* | 0.349 | *0.000* | 0.457 | 1.125 |
| Crop Rotation | 1.076 | *0.303* | 0.936-1.238 | 0.901 | 0.808-1.004 | *0.060* | 0.990 | 0.878-1.115 | *0.862* | 0.353 | *0.000* | 0.461 | 1.151 |
| Water Efficiency Irrigation | 0.621 | *0.000* | 0.478-0.807 | 0.859 | 0.771-0.957 | *0.006* | 1.000 | 0.689-1.452 | *1.000* | 0.367 | *0.000* | 0.454 | 1.147 |
| Agricultural Credit | 0.783 | *0.015* | 0.643-0.953 | 0.898 | 0.807-0.998 | *0.046* | 0.940 | 0.912-1.087 | *0.402* | 0.372 | *0.000* | 0.462 | 1.144 |
| Cash Other Farm | 1.652 | *0.000* | 1.454-1.877 | 0.890 | 0.794-0.997 | *0.045* | 1.022 | 0.921-1.135 | *0.676* | 0.295 | *0.000* | 0.509 | 1.098 |
| Cash Other Business | 0.818 | *0.002* | 0.719-0.932 | 0.864 | 0.776-0.963 | *0.008* | 1.136 | 1.018-1.267 | *0.023* | 0.389 | *0.000* | 0.446 | 1.134 |
| Informal Loan | 0.909 | *0.168* | 0.794-1.041 | 0.906 | 0.813-1.010 | *0.075* | 0.942 | 0.839-1.058 | *0.312* | 0.374 | *0.000* | 0.468 | 1.142 |
| Formal Loan | 0.701 | *0.000* | 0.584-0.842 | 0.909 | 0.818-1.011 | *0.079* | 0.837 | 0.721-0.972 | *0.019* | 0.377 | *0.000* | 0.454 | 1.132 |
| Cash Gifts | 1.027 | *0.706* | 0.895-1.178 | 0.867 | 0.776-0.968 | *0.011* | 1.144 | 1.020-1.283 | *0.022* | 0.360 | *0.000* | 0.467 | 1.145 |
| Group Participation | 0.722 | *0.000* | 0.633-0.823 | 1.095 | 0.970-1.235 | *0.141* | 0.723 | 0.649-0.806 | *0.000* | 0.431 | *0.000* | 0.469 | 1.096 |
| Female Household | 1.474 | *0.000* | 1.222-1.778 | 0.891 | 0.801-0.991 | *0.033* | 1.140 | 0.983-1.322 | *0.082* | 0.344 | *0.000* | 0.464 | 1.133 |
